# Landscape Ecology of Sylvatic Chikungunya Virus and Mosquito Vectors in Southeastern Senegal

**DOI:** 10.1371/journal.pntd.0001649

**Published:** 2012-06-12

**Authors:** Diawo Diallo, Amadou A. Sall, Michaela Buenemann, Rubing Chen, Oumar Faye, Cheikh T. Diagne, Ousmane Faye, Yamar Ba, Ibrahima Dia, Douglas Watts, Scott C. Weaver, Kathryn A. Hanley, Mawlouth Diallo

**Affiliations:** 1 Institut Pasteur de Dakar, Dakar, Senegal; 2 Departments of Geography and Biology, New Mexico State University, Las Cruces, New Mexico, United States of America; 3 Institute for Human Infections and Immunity, Center for Biodefense and Emerging Infectious Diseases, and Department of Pathology, University of Texas Medical Branch, Galveston, Texas, United States of America; 4 University of Texas, El Paso, Texas, United States of America; Duke University-National University of Singapore, Singapore

## Abstract

The risk of human infection with sylvatic chikungunya (CHIKV) virus was assessed in a focus of sylvatic arbovirus circulation in Senegal by investigating distribution and abundance of anthropophilic *Aedes* mosquitoes, as well as the abundance and distribution of CHIKV in these mosquitoes. A 1650 km^2^ area was classified into five land cover classes: forest, barren, savanna, agriculture and village. A total of 39,799 mosquitoes was sampled from all classes using human landing collections between June 2009 and January 2010. Mosquito diversity was extremely high, and overall vector abundance peaked at the start of the rainy season. CHIKV was detected in 42 mosquito pools. Our data suggest that *Aedes furcifer*, which occurred abundantly in all land cover classes and landed frequently on humans in villages outside of houses, is probably the major bridge vector responsible for the spillover of sylvatic CHIKV to humans.

## Author Summary

Chikungunya is a mosquito-borne virus that infects and sickens people in many tropical, urban regions of the world. This virus circulates in forest cycles of West Africa, where mosquitoes transmit it among non-human primates. It also infects humans via bridge vectors, mosquitoes that feed on both non-human primates and humans. To date, little is known about the environmental factors that influence the abundance and distribution of mosquito vectors that participate in the forest cycle of this virus or about specific mosquitoes that are likely to act as bridge vectors. We studied the distribution and abundance of mosquitoes potentially involved in the forest cycle in southeastern Senegal, as well as their infection by this virus. Satellite imagery was used to classify the region into the 5 most abundant land cover elements, and mosquitoes attracted to humans were collected in sites representing each land cover class. We found that *Aedes furcifer*, a mosquito that occurs in all land cover types and also enters villages to feed on humans, is probably the most important bridge vector between forest circulation and human populations.

## Introduction

Chikungunya virus (CHIKV, genus *Alphavirus*, family *Togaviridae*) is maintained in a sylvatic cycle in West Africa, where it is transmitted by a suite of sylvatic *Aedes* mosquito species among a group of reservoir hosts, including African green monkeys (*Chlorocebus sabaeus*), patas monkeys (*Erythrocebus patas*) and Guinea baboons (*Papio papio*), and possibly reservoir hosts in other orders of mammals [1]–[3]. Moreover, CHIKV has a history of emergence into humans followed by sustained human-to-human transmission, with the peridomestic mosquito *Aedes aegypti* serving as the primary vector [1], [3]. *Aedes albopictus* also serves as a vector of CHIKV in the human cycle. Indeed, this species, which originated from Asia, is a rapidly expanding exotic species in the Americas, Europe and Africa [3], [4] and was responsible for explosive CHIKV outbreaks in the Indian Ocean, Asia, Europe and Central Africa [1]–[6].

CHIKV infection results in an acute febrile disease accompanied by debilitating arthralgia that begins soon after infection but can persist for years [6]–[8]. CHIKV is usually confined to Africa and Asia. However recent transmission following the arrival of infected travelers has been observed in Europe [3] and there is considerable concern that CHIKV will invade the Americas, where both of its major peridomestic vectors are abundant and infected travelers have arrived from Asia and the Indian Ocean [9].

Although past studies have documented the ability of CHIKV to spill over from sylvatic habitats into humans in West Africa, little is known about the environmental factors that influence the risk of human infection or the participation of specific vector species in transmission from zoonotic reservoir hosts to humans. In eastern Senegal, amplifications of sylvatic CHIKV have been detected in mosquito pools in 1975, 1979, 1983, and 1992 in the Kédougou region. During these amplifications, CHIKV was isolated there from humans (one strain in 1975 and two strains in 1983) and monkeys (*Cercopithecus aethiops* in 1972, *Papio papio* in 1975 and *Erythrocebus patas* in 1983) [2], [10]. Following the 2003 amplification, a human outbreak of CHIKV occurred in 2004 in Kedougou among Peace Corps volunteers. In Western Senegal, three epidemics of CHIK fever have also been reported in 1966, 1982, and 1996 [2].

All of these data indicate frequent infection of humans by sylvatic CHIKV in southeastern Senegal. This transmission to humans may occur due to the movement of people into foci of infection in the forest, or to the movement of infected sylvatic vectors into areas occupied by humans. There is a low probability that humans are infected in the forest itself, as humans frequent the forest during daytime while the vectors described above are active at night. However, humans could be infected by sylvatic vectors in other biotopes that they enter at dusk or at night for farming purposes, or while commuting between their place of work and their village. Nonetheless, vector movement seems the more likely explanation for human infection, as dispersal of sylvatic *Aedes* vectors, particularly *Ae. furcifer*, into villages is well documented in Senegal [11], [12] and elsewhere in Africa [13].

In the current study, we sought to better understanding the environmental factors that influence the risk of human infection by CHIKV by rigorously testing the association between specific land cover elements and the abundance of *Aedes* vectors and of CHIKV infection of those vectors. We measured both the distribution and infection of vectors in multiple sampling plots within 5 different land cover classes (forest, savanna, barren, agriculture and village) and also the distribution and infection of these vectors within and among individual villages.

## Methods

### Study area

**Figure 1:**
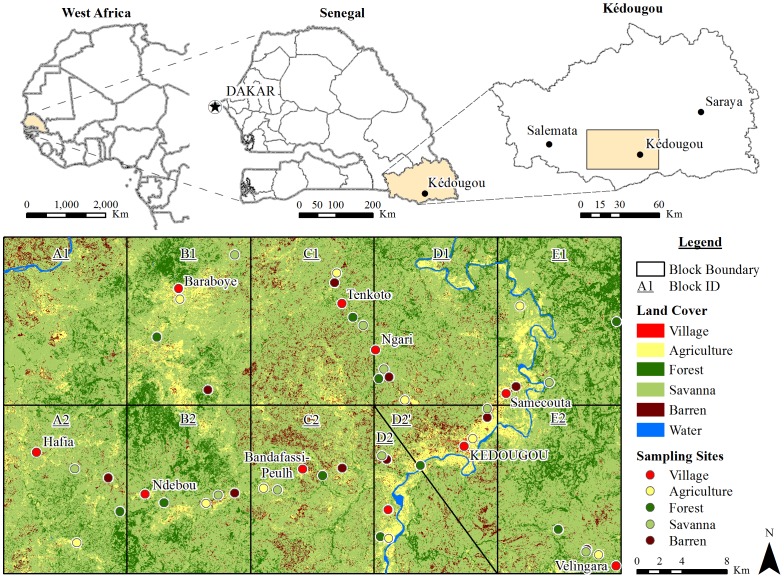
Location and land cover characteristics of study area. Symbols indicating sampling sites are centered around each site but are larger than the actual site in order to enhance visibility; thus some symbols overlap each other or the boundary of sampling blocks while actual sites do not overlap.

**Figure 2:**
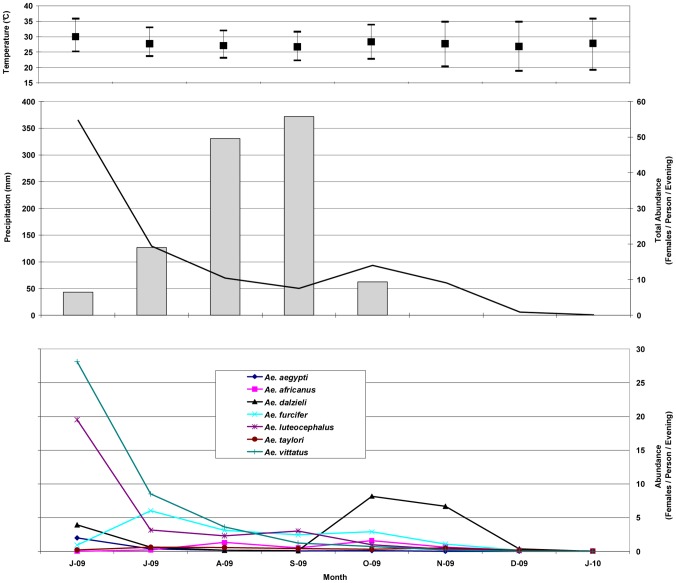
Meteorological conditions and abundance of potential CHIKV vectors between June 2009 and January 2001. The top panel shows mean temperature (solid square) bounded by maximum and minimum temperature (top and bottom bars) each month (www.worldclimate.com). The middle panel shows total precipitation (gray bars) (http://www.tutiempo.net/en/Climate/Kedougou/616990.htm), and total abundance of all sampled vector species (black line) per month. The bottom panel shows the monthly abundance of select mosquito species as indicated by the legend.

Our study was undertaken in the Kédougou Region of southeastern Senegal (12°33 N, 12°11 W) close to the borders of Mali and Guinea (Figure 1). The area (1,650 km^2^; 30 km in north-south and 55 km in east-west direction; center coordinates ∼12°36′N, 12°18′W) is located in the shield region of Senegal, with natural vegetation comprised of a mosaic of open savanna, woody savanna, outcrops of laterite (bowé), and relictual gallery forest, the latter concentrated along valleys and rivers [14]. Deforestation for cultivation and human habitations, as well as desertification, has greatly reduced the forested area, as in many other sub-Saharan regions of Africa. Characterized by a tropical savanna climate [15], the Kédougou region receives an average of 1,300 mm of total annual rainfall, with one rainy season from approximately May through November, and mean temperatures varying between about 25–33°C during the year (Figure 2)(http://www.worldclimate.com/). The human population of the region is ca. 80,000, of whom 55% are under the age of 20. It is primarily rural (84%) with a low density of inhabitants (4/km^2^), mostly living in small, dispersed villages averaging 60 inhabitants. The economy depends on horticulture and cattle farming, along with hunting, gathering and harvesting wood for crafts, necessitating human contact with forests. The primate fauna of the region includes three species, Guinea baboons, patas monkeys, and African green monkeys, which are known reservoir hosts of CHIKV [1], [2].

### Mosquito Sampling

**Figure 3:**
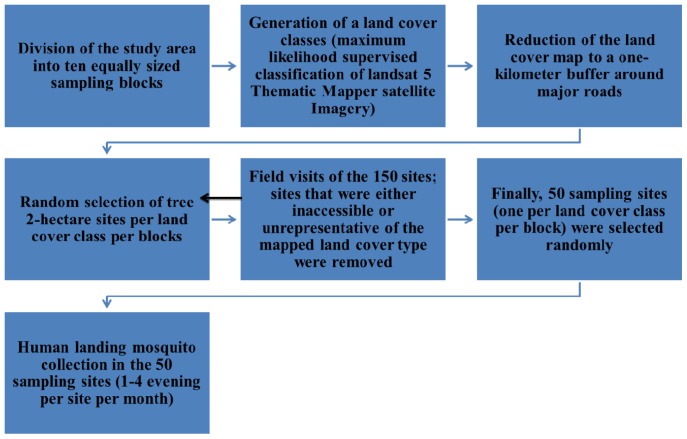
Chart showing the mosquito sampling strategy.

A six-stage sampling scheme, summarized in Figure 3, was used to identify ten sampling sites in each of the five predominant land cover classes (village, agriculture, barren, savanna, forest) in the study area. Stage I aimed at minimizing spatial autocorrelation among data collected in any given land cover type and entailed the division of the study area into ten equally sized sampling blocks (i.e., 5 north and 5 south of the central east-west line), each of which would eventually contain one representative sampling site per land cover class. In Stage II, a land cover map was generated by means of a maximum likelihood supervised classification of Landsat 5 Thematic Mapper satellite imagery acquired on June 11, 2009 (WGS Path 201/Row 51). Stage III entailed the extraction of only those areas from the land cover map that would likely be accessible in the field, and was accomplished by reducing the land cover map to a one-kilometer buffer around major roads.

In Stage IV, three 2-hectare sites were randomly selected within each of the five land cover classes (i.e., strata), within each of the 10 blocks, and within the one-kilometer buffer zone around major roads. Of the 150 sites, only one site per land cover and block was retained for mosquito sampling. However, three potential sites were identified initially because accessibility and land cover map accuracy in those specific sites were unclear prior to actual inspections. Stage V involved field visits of the 150 sites: sites that were accessible and representative of the mapped land cover type were retained for the final sampling site selection process (Stage VI); sites that were either inaccessible or unrepresentative of the mapped land cover type were removed from the pool of potential final sampling sites. As a result of Stage V endeavors, Block A1 had to be removed entirely from subsequent analyses due to inaccessibility. To avoid losing 5 sampling sites, Block D2—the most complex and centrally located block—was subdivided into two sub-blocks and Stages IV and V repeated in each. Finally, in Stage VI, one sampling site per land cover class per block was selected randomly from the pool of potential final sampling sites identified in Stage V.

Mosquitoes were sampled via human landing collections, the only effective method for sampling sylvatic *Aedes* and the most appropriate method for determining human risk of infection. Teams of three collectors working simultaneously in forest, savanna, agriculture, village and barren sites in a particular block from 6–9 PM, based on previous data on biting periodicity [12], collected all mosquitoes that landed on their legs. In each of the ten forest sites, mosquitoes were collected at ground level by 3 collectors. Additionally, in eight of the blocks (A2, B1, B2, C1, C2, D1, E1 and E2), a 9 m high platform was erected to enable collection by an additional 3 persons in the forest canopy. In each village, mosquito sampling was conducted by 6 landing collectors per evening. Five houses were selected in the village, following a transect going from one periphery to the opposite periphery via the center (one house in the center, one in each of the periphery sites, and one between each periphery and the center). Each sampling evening, one indoor and one outdoor collector were positioned at each house. On a given night, collectors would be set up at three houses on one half of the transect: one on the periphery, one at the middle point between the periphery and center, and one at the center. On the next night they were positioned on the opposite side to avoid bias due to possible vector confinement within villages. Sampling was performed monthly for 1 to 4 consecutive nights in each block.

### Mosquito identifications

At the end of each collection evening, mosquitoes were frozen and then sorted on a chill-table using morphological identification keys established by Edwards [16], Ferrara et al. [17], Huang [18], and Jupp [19] for the culicines and by Diagne et al. [20] for the anophelines. Mosquitoes were sorted into monospecific pools of up to 40 individuals and frozen in liquid nitrogen for virus detection attempts.

### Determination of Parity

The ovaries from a sample of the unengorged mosquitoes were dissected on a slide containing distilled water. The degree of coiling of ovarian tracheoles was then observed to determine whether the female was parous or nulliparous [21].

### Detection of virus in mosquito pools

To attempt virus isolation, monospecific mosquito pools were homogenized in 2.5 ml of Leibovitz 15 cell culture medium containing 20% fetal bovine serum (FBS) and centrifuged for 20 min at 10,000× g at 4°C. For each homogenate, 1 ml of the supernatant was inoculated into AP61 (*Ae. pseudoscutellaris*) or Vero African Green kidney cells as described previously [22]. Cells were incubated at 28°C (AP61) or 37°C (Vero), and cytopathogenic effects recorded daily. Within 10 d, slides were prepared for immunofluorescence assay (IFA) against 7 pools of immune ascitic fluids specific for most of the African mosquito-borne arboviruses. Viruses were identified by complement fixation and seroneutralization tests by intracerebral inoculation into newborn mice, as approved by the UTMB Institutional Animal Care and Use Committee.

For the real-time PCR assay, 100 µl of supernatant were used for RNA extraction with the QiaAmp Viral RNA Extraction Kit (Qiagen, Heiden, Germany) according to the manufacturer's protocol. RNA was amplified using real-time RT-PCR assay and an ABI Prism 7000 SDS Real-Time apparatus (Applied Biosystems, Foster City, CA) using the Quantitect kit (Qiagen, Hilden, Germany). The 25 µl reaction volume contained 1 µl of extracted RNA, 2x QuantiTect Probe, RT-Master Mix, 10 µM of each primer and the probe. The primer and probe sequences used those of Weidmann et al. (manuscript in preparation) for CHIKV, including the primers RP-CHIK (CCA AAT TGT CCY GGT CTT CCT) and FP-CHIK (AAG CTY CGC GTC CTT TAC CAA G) and the probe P-CHIK (6FAM –CCA ATG TCY TCM GCC TGG ACA CCT TT- TMR). The following thermal profile was used: a single cycle of reverse transcription for 10 min at 50°C, 15 min at 95°C for reverse transcriptase inactivation and DNA polymerase activation followed by 40 amplification cycles of 15 sec at 95°C and 1 min 60°C (annealing-extension step). Fluorescence was analyzed at the end of the amplification.

### Data Analysis

For analysis of the distribution of vector species among land cover classes, the average per site of female mosquitoes/person/evening (F/P/E) was used as a measure of absolute abundance. Abundance data were log transformed (log_10_ (n+1)) and analyzed using ANOVA followed by a Tukey-Kramer post-hoc test. In the case of *Ae. africanus*, there were too many zero values to conduct a valid ANOVA, so abundance data were recoded as present or absent in a designated site and compared using a contingency table analysis. Comparison of vector abundance between villages was conducted similarly. To analyze the distribution of each vector species in the periphery, middle and center of villages, the average abundance of a given species in each of the three regions of each of the 10 villages, collected outside of houses, was compared using ANOVA. For comparison of the abundance of all species in the periphery versus the center of the village, a paired t-test was used to compare the mean abundance, averaged across the 10 villages, of each of the 6 species at the periphery and center.

Spatial patterns of vector abundance were assessed using both global and local measures of spatial autocorrelation. At the global level, we quantified spatial autocorrelation with standard and cumulative spatial correlograms of Moran's I [23], i.e., graphs of Moran's I coefficients on the ordinate plotted against distance classes on the abscissa. We used eleven distance classes (0 to 5,000 m, 5,000 to 10,000 m, 10,000 to 15,000 m, etc. for the standard correlogram and 0 to 5,000 m, 0 to 10,000 m, 0 to 15,000 m, etc. for the cumulative correlogram), a compromise between Sturge's rule [24] and a straightforward lag distance, and an inverse distance weighting scheme. To test the significance of individual Moran's I coefficients at the 0.05 level, we used 9,999 permutations and a progressive Bonferroni correction to account for multiple testing. A correlogram was considered globally significant at the 0.05 level if at least one of the autocorrelation coefficients was significant at the Bonferroni-corrected level [25]. All Moran's I coefficients were computed using PASSaGE [26]. Moran's I values range from −1 (indicating dispersion) to +1 (indicating correlation). Negative values indicate negative spatial autocorrelation; positive values indicate positive spatial autocorrelation; a zero value indicates a random spatial pattern. At the local level, we quantified spatial autocorrelation with Anselin's [27] Local Indicators of Spatial Association (LISA) statistic using weights based on the four nearest neighbors, 9,999 permutations, and a 0.05 pseudo significance level.Statistically significant LISA statistics include two types of positive spatial autocorrelation (HH = High values surrounded by High values; LL = Low values surrounded by Low values) and two types of negative spatial autocorrelation (LH = Low values surrounded by High values; HL = High values surrounded by Low values

Parous and infection rates were compared using a contingency table analysis. The index of parous and biting was also calculated. Both analyses were conducted in *StatView 5.0* ® (SAS Institute, San Francisco, CA) or *JMP* ® (SAS Institute, Cary, N.C.). The pooled infection rate program (PooledInfRate, version 3.0, Center for Disease Control and Prevention, Fort Collins, CO: http://www.cdc.gov/ncidod/dvbid/westnile/software.htm) was used to calculate minimum field infection rates with a scale of 1,000 and the 95% confidence intervals for the species found positive for CHIKV.

## Results

### Mosquito abundance and distribution

Between June 2009 and January 2010, 39,799 mosquitoes were collected comprising 50 species within 6 genera (Table 1). Among host-seeking females of known or suspected CHIKV vectors, *Ae. vittatus* (22.98%), *Ae. furcifer* (18.66%), *Ae. dalzieli* (15.63%) and *Ae. luteocephalus* (13.05%) had the highest relative abundance and *Ae. taylori* (2.00%), *Ae. africanus* (1.71%) and *Ae. aegypti* (1.24%) had the lowest relative abundance. Absolute vector abundance showed considerable seasonal variation: *Ae. vittatus, Ae. luteocephalus* and *Ae. aegypti* reached their peak abundance in June at the beginning of the rainy season and declined drastically during the following months (Figure 2). Other species peaked twice between July and November 2009. Indeed, *Ae. africanus* exhibited 2 peaks of roughly equal level in August and October.

**Table 1:** Chikungunya virus infection rates among potential mosquito vectors of chikungunya virus, Kédougou, June 2009–January 2010.

Month	Block	Land cover class	Species	No. collected	Percentage (%)	No. pools tested	CHIKV Positive Pools	CHIKV Infection Rate (IR)	Infection rate 95% Lower Limit	Infection rate 95% Upper Limit
September	B2	Forest	*Ae. luteocephalus*	72		2	1	13.89	0.00	40.92
	C1	Forest	*Ae. luteocephalus*	51		3	1	19.61	0.00	57.66
	D1	Savanna	*Ae. luteocephalus*	2		1	1	500.00	0.00	1192.95
October	A2	Savanna	*Ae. hirsutus*	1		1	1	na	na	na
	B1	Barren	*Ae. furcifer*	56		1	1	17.86	0.00	52.54
	B2	Forest	*Ae. furcifer*	38		1	1	26.32	0.00	77.21
	C1	Forest	*Ae. taylori*	27		2	1	37.04	0.00	108.27
	C2	Forest	*Ae. africanus*	10		3	1	100.00	0.00	285.94
	D1	Forest	*Ae. furcifer*	138		5	3	21.74	0.00	46.07
	D1	Forest	*Ae. luteocephalus*	89		4	1	11.24	0.00	33.13
	E2	Forest	*Ae. aegypti*	2		2	1	500.00	0.00	1192.95
	E2	Forest	*Ae. africanus*	8		1	1	125.00	0.00	354.17
	D1	Village	*An. domicola*	2		2	1	500.00	0.00	1192.95
	D1	Village	*Ae. furcifer*	103		2	1	9.71	0.00	28.64
	E2	Village	*Ae. furcifer*	29		4	1	34.48	0.00	100.89
November	C1	Agriculture	*Ae. dalzieli*	57		2	1	17.54	0.00	51.63
	C1	Agriculture	*Ae. furcifer*	26		2	1	38.46	0.00	112.38
	C1	Forest	*Ae. furcifer*	14		2	1	71.43	0.00	206.33
	C1	Forest	*Ae. taylori*	27		2	1	37.04	0.00	108.27
	C1	Savanna	*Ae. dalzieli*	66		3	1	15.15	0.00	44.62
	C1	Savanna	*Ae. metallicus*	2		2	1	500.00	0.00	1192.95
	D1	Agriculture	*Ae. furcifer*	2		1	1	500.00	0.00	1192.95
	D1	Barren	*Ae. furcifer*	16		2	1	62.50	0.00	181.11
	D1	Forest	*Ae. furcifer*	69		2	1	14.49	0.00	42.69
	D1	Forest	*Ae. neoafricanus*	1		1	1	na	na	na
	D1	Forest	*Ae. taylori*	41		2	1	24.39	0.00	71.61
	E1	Barren	*Ae. centropunctatus*	1		1	1	na	na	na
December	A2	Forest	*Ae. dalzieli*	6		2	1	166.67	0.00	464.87
	A2	Forest	*Ae. furcifer*	12		2	1	83.33	0.00	239.71
	A2	Forest	*Ae. luteocephalus*	3		2	1	333.33	0.00	866.77
	A2	Forest	*Ae. taylori*	6		2	1	166.67	0.00	464.87
	B1	Forest	*Ae. dalzieli*	3		1	1	333.33	0.00	866.77
	B1	Village	*Ae. furcifer*	1		1	1	na	na	na
	C1	Agriculture	*Ae. taylori*	1		1	1	na	na	na
	D1	Barren	*Ma. uniformis*	3		2	1	333.33	0.00	866.77
	D1	Forest	*An. funestus*	2		2	1	500.00	0.00	1192.95
	D1	Forest	*Cx. poicilipes*	3		2	1	333.33	0.00	866.77
	D1	Savanna	*An. coustani*	8		2	1	125.00	0.00	354.17
	D1	Village	*Ae. furcifer*	3		2	1	333.33	0.00	866.77
**Totals**										
			*Ae. aegypti*	493	1.24	181	1	2.03	0.00	6.01
			*Ae. aegypti* male	8	0.02	6	0			
			*Ae. africanus*	682	1.71	40	2	2.94	0.00	7.00
			*Ae. centopunctatus*	68	0.17	28	1	14.71	0.00	43.32
			*Ae. dalzieli*	6219	15.63	338	4	0.64	0.01	1.27
			*Ae. furcifer*	7427	18.66	549	15	1.89	0.90	2.87
			*Ae. furcifer* male	86	0.22	63	1	11.63	0.00	34.29
			*Ae. hirsutus*	91	0.23	58	1	10.87	0.00	32.06
			*Ae. luteocephalus*	5194	13.05	363	5	0.96	0.12	1.81
			*Ae. metallicus*	186	0.47	80	1	5.38	0.00	15.89
			*Ae. neoafricanus*	1	0.00	1	1			
			*Ae. taylori*	795	2.00	163	5	6.29	0.79	11.78
			*Ae. taylori* male	74	0.19	50	0			
			*Ae. vittatus*	9147	22.98	589	0			
			*An. coustani*	1376	3.46	235	1	0.72	0.00	2.14
			*An. domicola*	22	0.06	14	1	45.45	0.00	132.50
			*An. funestus*	363	0.91	147	1	2.70	0.00	7.97
			*Cx. poicilipes*	51	0.13	30	1	19.23	0.00	56.56
			*Ma. uniformis*	1315	3.30	116	1	0.74	0.00	2.18
			other mosquitoes*	6201	15.58	1160	0			
			Total	39799	100	4211	0			

*: Others mosquitoes: Ae. argenteopunctatus, Ae. cozi, Ae. cumminsii, Ae. fowleri, Ae. mcintoshi, Ae. minutus, Ae. mixtus, Ae. ochraceus, Ae. unilineatus, Ae. vexans, An. brohieri, An. flavicosta, An. gambiae, An. hancocki, An. nili, An. pharoensis, An. pretoriensis, An. rufipes, An. squamosus, An. wellcomei, An. ziemanni, Cx. annulioris Cx. antennatus, Cx. bitaeniorhynchus, Cx. ethiopicus, Cx. macfiei, Cx. neavei, Cx. nebulosus, Cx. perfuscus, Cx. quinquefasciatus, Cx. tritaeniorhynchus, Eretmapodites quinquevittatus, Ma. africana, Urotaenia mayeri.

The patterns of precipitation and temperature over the mosquito sampling period are shown in Figure 2. With a total precipitation of 1087.3 mm (http://www.tutiempo.net/en/Climate/Kedougou/616990.htm), 2009 had a lower rainfall compared to the average of 1263 mm between 1967 and 1990 (www.worldclimate.com). Total vector abundance peaked at the start of the rains in 2009 in June and declined thereafter as rainfall increased and temperature decreased. However there was a second, albeit much smaller peak in November as rainfall dropped off abruptly and temperatures began to climb.

Potential sylvatic CHIKV vectors also showed significant variation in their distributions among land cover classes (Table 2). All species were collected in all land cover classes, with the notable exception of *Ae. africanus*, which was absent from barren, agricultural and indoor village sites. A contingency table analysis showed a significant difference in the distribution of *Ae. africanus* among land cover classes (χ2 = 25.9, df = 6, P = 0.0001); results of the remaining statistical comparisons of absolute abundance are listed in Table 2.

**Table 2:** Abundance in different land cover classes near Kédougou, Senegal, of potential chikungunya virus mosquito vectors.

Abundance† (Females/Person/Evening)									
	Classes	*Ae. africanus*	*Ae. luteocephalus*	*Ae. taylori*	*Ae. aegypti*	*Ae. vittatus*	*Ae. dalzieli*	*Ae. furcifer*	Main vectors
Land cover	Forest-canopy	3.49±0.59^a^	8.31±0.92^a^	2.07±0.20^a^	0.05±0.02^b^	0.50±0.20^c^	0.29±0.08^cd^	4.15±0.39^ab^	17.62±1.26^a^
	Forest-ground	1.00±0.19^b^	4.12±0.39^a^	0.86±0.12^ab^	0.30±0.05^a^	2.53±0.36^abc^	2.12±0.32^abcd^	2.80±0.24^ab^	13.18±0.77^a^
	Savannah	0.01±0.01^b^	0.71±0.10^b^	0.22±0.04^ab^	0.19±0.04^ab^	4.34±0.46^ab^	4.64±0.71^ab^	2.66±0.34^ab^	12.77±0.88^a^
	Barren	0.00±0.00^b^	0.35±0.06^b^	0.12±0.03^b^	0.05±0.01^b^	6.56±0.61^a^	3.37±0.53^abc^	3.02±0.30^ab^	13.47±0.89^a^
	Agriculture	0.00±0.00^b^	0.40±0.10^b^	0.09±0.03^b^	0.14±0.03^ab^	5.66±0.58^a^	4.36±0.51^a^	2.73±0.30^ab^	13.38±0.85^a^
	Village-indoor	0.00±0.00^b^	0.10±0.05^b^	0.01±0.01^b^	0.07±0.02^b^	0.89±0.17^bc^	0.32±0.08^d^	1.13±0.16^a^	2.52±0.31^d b^
	Village-outdoor	0.01±0.01^b^	0.25±0.09^b^	0.03±0.01^b^	0.23±0.03^ab^	2.81±0.39^abc^	0.98±0.19^bcd^	4.37±0.38^ab^	8.67±0.65^a^
	F	NA	17.23	7.23	4.15	6.35	6.66	2.13	8.18
	df	NA	6; 61	6; 61	6; 61	6; 61	6; 61	6; 61	6; 61
	P	NA	<0.0001	<0.0001	0.0015	<0.0001	<0.0001	0.062	<0.0001
Village, position	Village-Periphery		0.42±0.38^a^	0.15±0.15^a^	0.67±0.46^a^	3.02±1.94^a^	1.36±0.47^a^	4.87±1.20^a^	
	Village-Middle		0.20±0.13^a^	0.00±0.00^a^	0.18±0.07^a^	1.98±0.75^a^	0.45±0.24^a^	3.47±0.79^a^	
	Village-Center		0.11±0.05^a^	0.03±0.03^a^	0.25±0.06^a^	1.74±0.76^a^	0.63±0.35^a^	2.59±0.74^a^	
	df		2; 27	2; 27	2; 27	2; 27	2; 27	2; 27	
	F		0.47	0.59	0.95	0.28	1.74	1.51	
	P		0.62	0.56	0.4	0.76	0.2	0.24	

**†:** For each species, means that do not share a superscript letter are significantly different by a Tukey-Kramer post-hoc test, P≤05, excepting *Ae. africanus*, which was analyzed by contingency table analysis and pairwise Fisher's exact tests due to the large numbers of 0's in the dataset; see text for data.

Importantly, all of the mosquito species showed significant differences in absolute abundance among land cover classes except for *Ae. furcifer*, which showed similar abundance in all five classes (F = 2.13, df = 6, 61, P = 0.062). This species had it highest abundances in the forest-canopy and the village-outdoor. However *Ae. furcifer* preferred the village outdoor environment, and was significantly more abundant outdoors than indoors in villages. Moreover, compared to the others vectors, it also had the highest abundance in village environment both outdoor and indoor. Indeed, the ratio of the abundance of *Ae. furcifer* to *Ae. dalzieli* and *Ae. taylori* in village-outdoor was 4.5:1 and 146.0:1, respectively.

*Aedes africanus, Ae. luteocephalus* and *Ae. taylori* were most abundant in the forest, particularly in the forest canopy. *Aedes aegypti* was most abundant in the forest at ground level. *Aedes vittatus* was most abundant in barren, agricultural and ground level forest sites while *Ae. dalzieli* was most abundant in savannah. The global abundance of CHIKV vectors was comparable across all land cover classes but was significantly lower inside of houses in villages than in any other sites.

**Figure 4:**
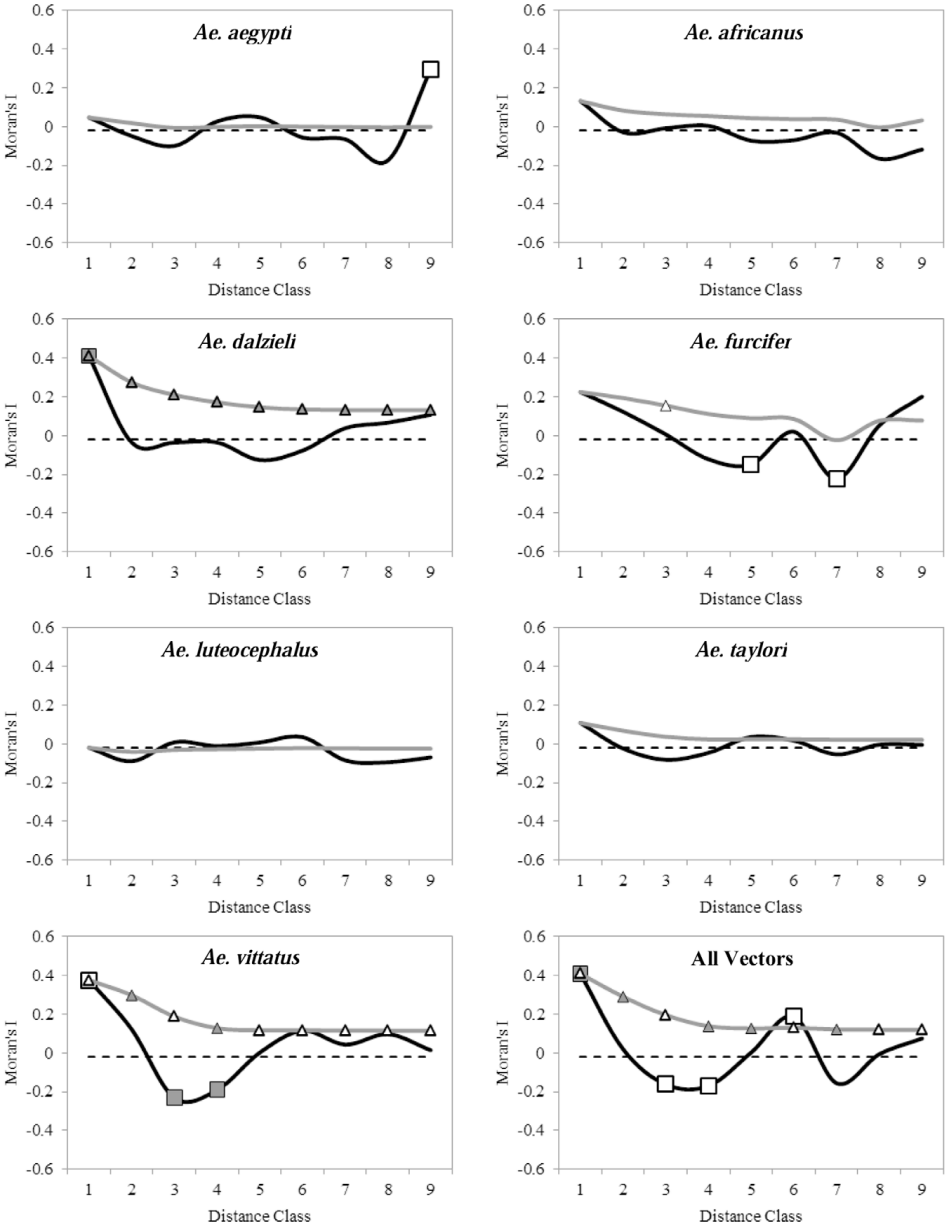
Standard (solid black line) and cumulative (solid gray line) Moran's I spatial correlograms. Solid squares/triangles indicate spatial autocorrelation statistics that remain significant after progressive Bonferroni correction; white squares/triangles indicate statistics that were significant before the correction and non-significant afterwards. The dashed line indicates the expected value of Moran's I under the null hypothesis of no spatial autocorrelation (here: 0.02041). Spatial autocorrelation coefficients for distance classes 10 and 11 are not shown here, because they only include the pairs of study area border point locations and less than 2% of all pairs considered.

**Figure 5:**
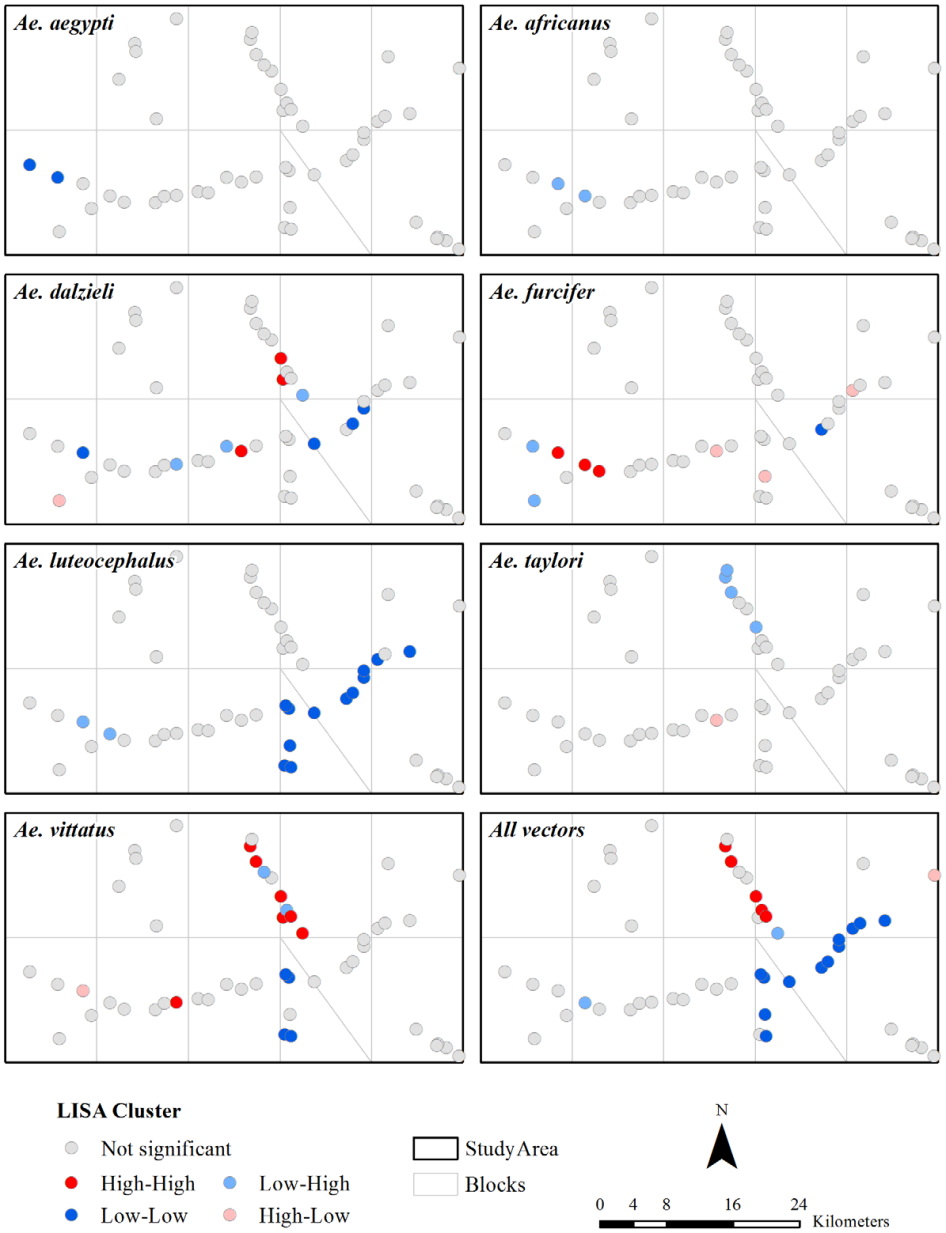
LISA maps of vector abundance. The analyses were based on 9,999 permutations and a pseud significance value of 0.05.

As shown in Figure 4, the spatial correlograms of *Ae. aegypti*, *Ae. africanus*, *Ae. furcifer*, *Ae luteocephalus*, and *Ae taylori* were not significant (p>0.05), indicating that the abundance of these vectors exhibited no global spatial autocorrelation. *Ae. dalzieli* exhibits significant positive spatial autocorrelation only in the first distance class and *Ae. vittatus* significant negative spatial autocorrelation in distance classes 3 and 4. The standard correlogram for the abundance of all vectors suggests significant positive spatial autocorrelation in the first distance class; spatial autocorrelation in subsequent classes is not significant at the Bonferroni-corrected significance level. The cumulative correlograms suggest that most of the spatial autocorrelation in our vector abundance data occured in the first lag (0 to 5,000 m). The vectors with no global spatial autocorrelation generally exhibited the least amount of local spatial autocorrelation (Figure 5). *Ae. aegypti* exhibited some positive spatial autocorrelation (LL: A2 urban and A2 savannah), *Ae. africanus* some negative spatial autocorrelation (LH: A2 barren and B2 urban), *Ae. furcifer* mostly positive spatial autocorrelation (HH: A2 barren, B2 urban, and B2 forest), and *Ae taylori* mostly negative spatial autocorrelation (LH: in Block C1). *Aedes luteocephalus* showed very notable clusters of positive spatial autocorrelation (LL in Blocks D2 and D2′) and *Ae. vittatus* has mostly positive spatial autocorrelation (HH in Blocks C1 and D1 and LL in Block D2). The LISA map for abundance of all vectors (Figure 5) showed that, when combined, there was essentially no local negative spatial autocorrelation. Spatial autocorrelation in the western half of the study area was mostly non-significant. Positive spatial autocorrelation clusters were quite common, with hot spots (HH clusters) limited to the northern half (Blocks C1 and D1) and cold spots (LL clusters) to the east/southeast (Blocks D2, D2′, and E1).

The majority of mosquitoes dissected were parous for all species (Tables 3 and 4). However, *Ae. africanus* showed the highest parous rate (P<0.0001), while *Ae. vittatus* had the lowest. The monthly parous rates of each vector, except *Ae. africanus* (P = 0.06), were significantly different and the highest rates were observed in October, November and December, when almost all females were parous (Table 3). The index of parous rate/biting rate increased from August to December except for a drop in November for *Ae. taylori*. All the vectors except *Ae. furcifer*, *Ae. vittatus* and *Ae. luteocephalus* had high and statistically comparable parous rates in the different land cover classes (Table 4; P>0.1). The highest parous rates for both *Ae. furcifer* (P = 0.02) and *Ae. vittatus* (P = 0.02) were in the village sites and the highest rates for *Ae. luteocephalus* were in the savanna and village sites (P = 0.06).

**Table 3:** Temporal dynamics of parous rates (PR, the number parous/number dissected) and index of PR/biting rate (BR) in potential mosquito vectors of chikungunya virus, Kédougou, 2009.

	August	September		October		November		December		
Species	PR	Index (PR/BR)	PR	Index (PR/BR)	PR	Index (PR/BR)	PR	Index (PR/BR)	PR	Index (PR/BR)
*Ae. aegypti*	62.1 (18/29)	460	0 (0/2)	0	100 (13/13)	1176.5	100 (6/6)	4166.7	100 (3/3)	10000
*Ae. africanus*	91.7 (22/24)	69.9	-	-	100 (19/19)	63.7	100 (48/48)	163.9	100 (27/27)	714.3
*Ae. furcifer*	59 (177/300)	12.8	64 (48/75)	28.2	95.0 (113/119)	30.5	100 (266/266)	68.0	100 (45/45)	384.6
*Ae. luteocephalus*	72.8 (123/169)	19.3	85.1 (40/47)	23.3	88.9 (24/27)	55.9	100 (77/77)	163.9	100 (25/25)	625.0
*Ae. taylori*	67.9 (57/84)	123.5	84.6 (11/13)	228.6	76.9 (20/26)	291.4	100 (126/126)	153.8	100 (45/45)	344.8
*Ae. vittatus*	57.0 (154/270)	11.7	61.4 (35/57)	63.3	81.6 (40/49)	127.5	100 (72/72)	370.4	100 (8/8)	2500

Nb: % (Parous rate), No. p (Number parous), No. d (Number dissected), br (Biting rate).

**Table 4:** Parous rates (number parous/number dissected) in different land cover classes of potential mosquito vectors of chikungunya virus, Kédougou, 2009.

Species	Forest	Savanna	Barren	Agriculture	Village	Total
*Ae. aegypti*	75.0 (18/24)	33.3 (2/6)	100 (1/1)	88.9 (8/9)	84.6 (11/13)	75.5 (40/53)
*Ae. africanus*	98.3 (115/117)	na	na	na	100 (1/1)	98.3 (116/118)
*Ae. furcifer*	78.7 (211/268)	77.3 (99/128)	78.4 (105/134)	77.3 (75/97)	89.3 (159/179)	80.5 (649/806)
*Ae. luteocephalus*	83.4 (211/253)	97.3 (36/37)	77.8 (14/18)	70.4 (19/27)	90.0 (9/10)	83.8 (289/345)
*Ae. taylori*	88.7 (219/247)	76.2 (16/21)	85.7 (6/7)	100 (15/15)	75.0 (3/4)	88.1 (259/294)
*Ae. vittatus*	69.5 (66/95)	63.2 (60/95)	59.7 (71/119)	72.2 (57/79)	80.9 (55/68)	67.8 (309/456)

Within villages, 5,573 mosquitoes were collected, representing 38 species within 6 genera; Table 2 shows absolute abundance of these species. *Aedes furcifer* (34.7% of the mosquitoes collected), *Ae. vittatus* (25.4%), *Ae. minutus* (13.1%), *Ae. dalzieli* (8.5%), *Culex quinquefasciatus* (5.9%), *Ae. luteocephalus* (2.4%) and *Ae. aegypti* (3.1%) had the highest relative abundance. *Aedes taylori*, representing only 0.3% of the mosquitoes collected, had the lowest relative abundance within the villages. None of the individual species differed significantly in their absolute abundance in the periphery, middle and center of villages (Table 2). However, when mean abundance of each of the six species of mosquitoes was compared at village periphery versus center, abundance was found to be significantly higher at the periphery (paired t-test, df = 5, t = 2.6, P = 0.048).

**Figure 6:**
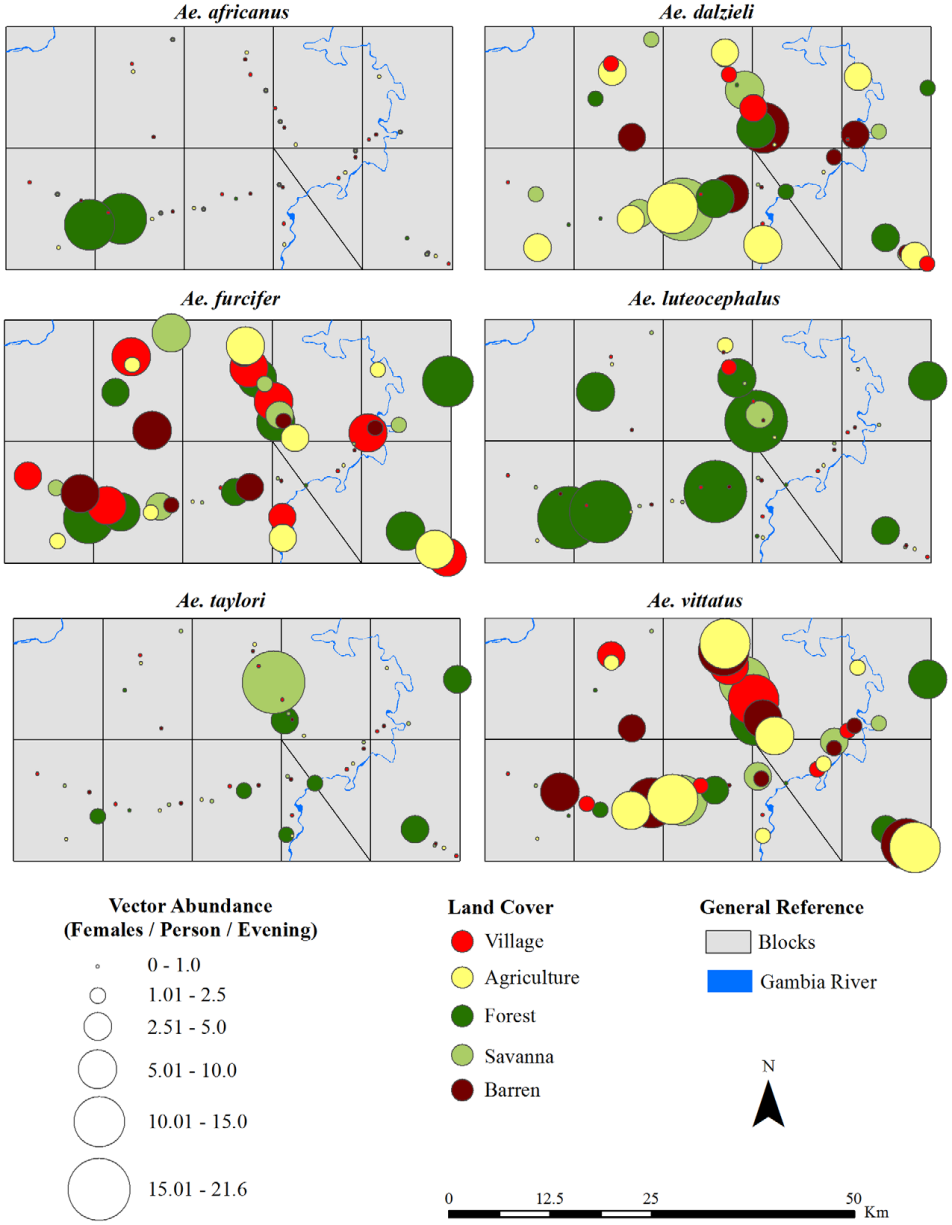
Absolute abundance of potential CHIKV vectors at each site between June 2009–January 2010. The size of each symbol indicates abundance at each site as indicated in the legend; color indicates the land cover class in which the mosquitoes were collected as indicated in the legend.

Large and statistically significant differences in absolute vector abundance were observed among villages (Figure 6). *Aedes africanus* and *Ae. taylori* had low abundance and were collected at one village (E1) and 5 of the 10 villages (B1, B2, C1, E1 and E2), respectively. Absolute abundance of *Ae. vittatus* and *Ae. dalzieli* were highest in the village in block D2′, while absolute abundance of *Ae. aegypti* was highest in the village in block D1 and that of *Ae. luteocephalus* was highest in the villages in blocks C1 and B1. *Aedes furcifer* was least abundant in villages in blocks C2 and D2′. In total, potential CHIKV vectors were present at all villages but were most abundant at the village in D1 (Ngari) and least abundant at villages C2 and D2′.

### Detection of virus in mosquito pools

CHIKV was detected in 42 of the 4,211 mosquito pools collected from June, 2009 to January, 2010. Table 1 lists the number of pools and CHIKV infection rates of mosquito species. The 42 infected pools were distributed as follows: *Ae. furcifer* (15 pools of females and 1 of males), *Ae. taylori* (5 female pools), *Ae. dalzieli* (4 female pools), *Ae. luteocephalus* (5 female pools), *Ae. africanus* (2 female pools) and *Ae. aegypti*, *Ae. metallicus*, *Ae. neoafricanus*, *Ae. centropunctatus*, *Ae. hirsutus*, *An. domicola*, *An. funestus*, *An. coustani*, *Mansonia uniformis* and *Cx. poicilipes* (1 female pool each) captured in September, October, November and December. No CHIKV was detected in mosquitoes collected in the other months. These data represent the first detection of CHIKV in *Ae. metallicus*, *Ae. centropunctatus*, *Ae. hirsutus*, *An. domicola*, and *Cx. poicilipes*, and the first observation of CHIKV in a male *Ae. furcifer* from Senegal. Mean infection rates among species differed significantly (P<0.05). Higher and statistically comparable infection rates were observed in *Ae. furcifer* males, *Ae. taylori*, *Ae. centropunctatus*, *Ae. metallicus*, *Ae. hirsutus*, *An. domicola* and *Cx. poicilipes* females (P = 0.48). Taking into account the temporal dynamics of CHIKV, the highest infection rates were those of *An. domicola* in October, *Ae. centropunctatus* in November and *Ae. furcifer* males in December. Detailed characterization of the CHIKV isolates and sequences will be described separately.

CHIKV infection rates showed temporal and spatial variation. They were higher in December for *Ae. furcifer*, *Ae. luteocephalus Ae. taylori* and *Ae. dalzieli*. The differences were statistically significant except for *Ae. taylori* (P = 0.42) and *Ae. luteocephalus* (P = 0.2). CHIKV was detected from mosquitoes collected in 8 of 10 blocks (A2, B1, B2, C1, C2, D1, E1, E2) and in all land cover classes (Table 1), including 7 forest (24 pools), 3 savanna (5 pool), 3 barren (pools), 2 agricultural (4 pools) and 3 village (5 pools) sites. To assess variation among land cover classes, each site was coded as positive (at least one CHIKV-positive pool) or negative (no CHIKV-positive pools). Based on this coding, there was no significant association between land cover class and presence of CHIKV (χ2 = 8.0, df = 4, P = 0.09). However, there was a significant difference among blocks (χ2 = 17.7, df = 9, P = 0.04), with CHIKV being detected in all land cover sites in block D1, no land cover sites in blocks D2 and D2′, and some but not all sites in the remaining blocks. There was a significant, positive correlation between total vector abundance and the number of CHIKV-positive pools across sites (Spearman rank correlation, N = 50, P = 0.003).

## Discussion

The mosquito fauna of the Kédougou region is very diverse. Since the initiation of entomological studies in the area, over 102 species belonging to more than 7 genera have been collected [2], [11], [28], [29]. This high diversity is due to the availability of a wide variety of larval habitats (such as clean slow-running streams and ponds, temporary and semi-permanent pools, and small water collections on the ground or phytotelmata), vertebrate hosts, nectar sources, resting and mating places. However, the amount of diversity detected varies widely among studies, depending on specific sampling methods used (human landing collections alone or with animal baited trap, and larval sampling) and the time period and area covered.

The goal of this study was to determine when and where humans may be exposed to sylvatic CHIKV infection and to identify the bridge vectors responsible for such spillover. To accomplish this, we measured the relative abundance and parity of all putative vectors across different land cover classes at the onset of, during, and immediately after the rainy season. Additionally we conducted detailed sampling within villages to assess exposure to vectors inside versus outside of houses and at the center versus the periphery of villages. The study was specifically designed to avoid spatial autocorrelation by random selection of sampling sites within larger sampling blocks, and as expected we detected minimal levels of such autocorrelation. We collected few potential CHIKV vectors inside houses, indicating an exophagic feeding behavior of these mosquitoes. However, these vectors actively sought human hosts in all land cover classes investigated. In the evening, when the vectors peak in landing rates [11], [30], humans are generally within villages, suggesting that most exposures to sylvatic arboviruses occurs within villages in this region. Additionally, the majority of mosquitoes we collected were parous, indicating that they were in their second or a subsequent gonotrophic cycle and thus had high vectorial capacity. The season increase in the index of parous rate/biting rate suggests little or no recruitment of new mosquitoes to the biting population in October, November and December. Parous rates of vectors were higher in villages than other land cover classes, so humans are at risk of being infected by sylvatic CHIKV in every type of land cover we sampled, but are at greatest risk while outside of houses within villages. Across all species, vector abundance was higher at the periphery of villages than in the center, suggesting that vectors invade villages from surrounding land cover types and that risk of infection may therefore be highest at the edges of villages.

The unexpectedly high host seeking activity of mosquitoes in land cover classes where their known, preferred hosts (humans and monkeys) are not generally present, such as barren areas, suggests that they probably feed on other crepuscular or nocturnal vertebrates. These other species could also be involved in undocumented enzootic cycles of CHIKV in the Kédougou area, as has been suggested by associations of CHIKV with birds, bats and other mammals in Africa [2], [31], [32], [33].A more comprehensive understanding of the enzootic ecology of this virus in the region will require the identification of other potential vertebrate hosts and the description of their roles in the sylvatic cycle of CHIKV. Collection and identification of bloodmeals from feral, engorged vectors will be necessary to achieve this objective.

We associated five mosquito species with CHIKV for the first time. These new associations may reflect the wide spatial and seasonal scope of our study, since all the previous studies of CHIKV in the Kédougou area focused on only one forest-gallery site and a few villages. Detection of CHIKV from a male *Ae. furcifer* in the Kédougou region during our investigation, and in Ivory Coast [34], may suggest vertical transmission of this virus. Dengue and yellow fever viruses have also been detected in male *Ae. furcifer* and *Ae. furcifer-taylori* in Kédougou in previous studies [11], [35].The ecology of sylvatic *Aedes* mosquitoes in Africa has been well studied because of their role in the transmission of yellow fever virus [30], [35]. We demonstrated that the distribution of some vector species, such as *Ae. luteocephalus*, *Ae. taylori* and *Ae. africanus*, was largely restricted to the forest canopy. This observation is consistent with most similar studies in East and West Africa [36], [37], although *Ae. africanus* was collected within human settlements and inside houses in southeastern Nigeria [38], [39]. In combination with data suggesting that these mosquitoes feed only during the evening [12], [30], our data suggest that these exophilic species are primarily involved in the maintenance of the zoonotic, sylvatic cycle of CHIKV with little impact on spillover into humans.

*Aedes furcifer*, in contrast, had high and comparable abundance in the forest canopy and in villages outside houses. It was the only species that frequently contacted humans in villages, corroborating previous observations [11], [40]. Abundance of this species differed significantly among villages and occurred at lowest density in the two most developed of the ten villages we studied. This species is also the only one of the putative sylvatic vectors that is commonly infected with sylvatic arboviruses within villages in the area [11], [40]. Thus it is likely that *Ae. furcifer* is the principal vector for spillover of sylvatic arboviruses into humans in this area. However, the extreme generalism of *Ae. furcifer* for different land cover classes is unusual, and we caution that investigation of the population genetics of this species is warranted before firm conclusions can be made about its role as spillover vector.

The fact that the CHIKV was detected in 3 of the 10 villages, and that the distribution of CHIKV was significantly different among sampling blocks, suggests that the risk of transmission to humans may be localized or spatially or temporally heterogeneous. These findings also suggest the need to further characterize the different land cover classes in order to identify subclasses that could differ among blocks. Vector abundance showed a positive correlation with the number of CHIKV-positive pools detected at a site, but vector density may not be the only explanation for variation in the distribution of CHIKV, and therefore this phenomenon merits further study. For example, these three villages in which CHIKV was detected are the closest to gallery forests of the ten villages studied.

Although *Ae. dalzieli* and *Ae. vittatus* were widely distributed within the study area (in forest floor, savanna, barren and agricultural sites), and had high abundance in some villages, they have never been found infected with CHIKV within villages in the Kédougou area. Thus, these two species could be involved in virus dissemination from the forest to other land cover classes and could also play a role in potential secondary transmission cycles of the virus among as-yet unidentified species, but are unlikely to be important for spillover of sylvatic CHIKV. *Aedes aegypti* showed low human landing rates in all land cover classes. Previous studies have also found that *Ae. aegypti* did not land on humans in high numbers in the Kédougou area [11], [12]. The low abundance of human-seeking *Ae. aegypti* females despite high larval population density of this species in villages is probably due to its zoophilic tendency in West Africa [41], [42]. Indeed, only the sylvatic form, *Ae. aegypti* subspecies *formosus*, occurs in the Kédougou area [43], and this subspecies is thought to feed mainly on wild animals other than primates. Thus, although *Ae. aegypti aegypti* is the main CHIKV epidemic vector worldwide [1], [8], [44], *Ae. aegypti* formosus probably plays no major role in either maintenance of sylvatic cycle or spillover to humans in this area.

In summary, our data give new insight into the temporal and spatial dynamics of the extraordinarily diverse guild of sylvatic CHIKV mosquito vectors in an area where, at regular intervals, this virus undergo amplifications in their animal reservoirs that result in spillover infection of humans. While many vectors may participate in maintenance of sylvatic CHIKV, *Ae. furcifer* is most likely to be responsible for spillover into humans due to its broad land cover preferences and rates of human contact within village perimeters. This information can be used to inform the local population of the places and times of greatest risk for exposure so that mosquito avoidance or protective measures can be implemented. The detection of CHIKV-infected mosquito pools only during the rainy season was expected, but the aggregation of infected pools in specific sampling blocks, rather than in particular land cover classes, was not. We recognize that limited sampling for only a few hours per day and during only one year could have resulted in some anomalous findings or biased results. Additional surveillance and further analysis will be needed to reveal the ecological factors that shape the distribution of CHIKV; our surveillance efforts in Kédougou are ongoing to accomplish this goal.
